# Potential Biomarker for Human Uterine Leiomyosarcoma

**DOI:** 10.14740/jocmr1867e

**Published:** 2014-07-28

**Authors:** Takuma Hayashi, Akiko Horiuchi, Hiroyuki Aburatani, Osamu Ishiko, Nobuo Yaegashi, Yae Kanai, Dorit Zharhary, Tanri Shiozawa, Susumu Tonegawa, Ikuo Konishi

**Affiliations:** a Department of Immunology and Infectious Disease, Shinshu University School of Medicine, Matsumoto, Nagano 390-8621, Japan; b Promoting Business Using Advanced Technology, Japan Science and Technology Agency (JST), Chiyoda, Tokyo 102-8666, Japan; c SIGMA-Aldrich Collaboration Laboratory; d Horiuchi Ladies Clinic, Matsumoto, Nagano 390-0821, Japan; e The Cancer System Laboratory, Research Center for Advanced Science and Technology, The University of Tokyo, Meguro, Tokyo 153-9804, Japan; f Department of Obstetrics and Gynecology, Osaka City University Graduate School of Medicine, Osaka, Osaka 545-8585, Japan; g Department of Obstetrics and Gynecology, Tohoku University Graduate School of Medicine, Sendai, Miyagi 980-8574, Japan; h Pathology Division, National Cancer Center Research Institute, Chuoku, Tokyo 104-0045, Japan; i The International Human Epigenome Consortium (IHEC) and CREST, Japan Science and Technology Agency (JST), Chiyoda, Tokyo 102-8666, Japan; j Sigma-Aldrich Israel Ltd, Rehovot 76100, Israel; k Department of Obstetrics and Gynecology, Shinshu University School of Medicine, Matsumoto, Nagano 390-8621, Japan; l Picower Institution and Department of Biology, Massachusetts Institute of Technology, Cambridge, MA 02139-4307, USA; m Department of Obstetrics and Gynecology, Kyoto University Graduate School of Medicine, Kyoto, Kyoto 606-8507, Japan

## To the Editor

Sarcomas are a rare form of malignant tumor, with less than 15,000 new cases being diagnosed each year in the United States. In spite of their rarity, sarcomas are highly debilitating malignancies that are often associated with significant morbidity and mortality. They are also biologically very heterogeneous because they originate from many different tissues and cell types. Sarcomas have classically been defined by their tissue of origin and are additionally stratified according to their histopathology or the age of the patient at diagnosis. Uterine mesenchymal tumors that develop in the myometrium have traditionally been divided into benign uterine usual LMA, cellular LMA and malignant Ut-LMS based on cytological atypia, mitotic activity and other criteria. Ut-LMS is relatively rare, having an estimated annual incidence of 0.64 per 100,000 women [1], and is resistant to chemotherapy and radiotherapy; therefore, surgical interventions are virtually the only means of treatment [2, 3]. The prognosis of patients with Ut-LMS is poor, and the 5-year survival rate is approximately 35%. Uterine LMA may occur in 70-80% of women by the age of 50 years [4]. Difficulties have been reported in distinguishing Ut-LMS from other uterine mesenchymal tumors including uterine LMA, and a diagnosis generally requires surgery and cytoscopy. Diagnostic categories for uterine mesenchymal tumors and morphological criteria are used to assign cases. The non-standard subtypes of uterine mesenchymal tumors such as the epithelioid and myxoid types are classified in a different manner using these features; therefore, a diagnostic method needs to be established that can identify non-standard smooth muscle differentiation [5, 6].

High estrogen levels have been shown to significantly influence the development of tumors in the uterine body [7]. However, the molecular mechanisms underlying the transformation of uterine LMA and development of Ut-LMS remain unknown. Tumors that have developed and grown in the myometrium increase in size due to the influence of the female hormone, estrogen, which leads to the generation of more tumors. However, a relationship has not yet been reported between the development of Ut-LMS and hormonal conditions, and no obvious risk factors have been identified. The identification of risk factors associated with the development of human Ut-LMS will contribute significantly to the development of preventive and therapeutic treatments. Cytoplasmic proteins are mostly degraded by a protease complex referred to as the 20S proteasome, which has many substrates that consist of twenty-eight 20 to 30 kDa subunits [8, 9]. Proteasomal degradation is essential for many cellular processes, including the cell cycle, regulation of gene expression and immunological function [10]. A previous study reported that an interferon (IFN)-γ treatment induced the expression of large numbers of responsive genes, the β-ring subunits of proteasomes, i.e., low-molecular mass polypeptide (LMP)2/β1i, LMP7/β5i and LMP10/multicatalytic endopeptidase complex-like (MECL)-1/β2i [11]. Ut-LMS was detected in female LMP2/β1i-deficient mice at 6 months or older, and its incidence at 14 months was approximately 40% [12]. Histopathological studies of LMP2/β1i-lacking uterine tumors have revealed the characteristic abnormalities of Ut-LMS [12].

**Figure 1:**
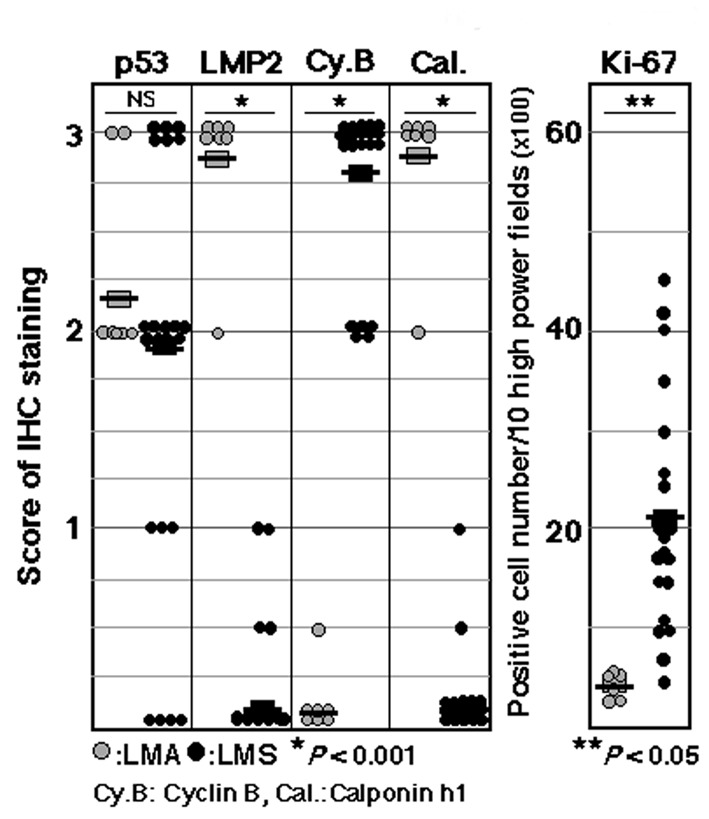
Summary of IHC experiments for p53, LMP2, cyclin B, calponin h1 and ki-67 expression levels in leiomyoma (LMA) and leiomyosarcoma (LMS). IHC experiments were performed using seven LMA tissue samples and 23 LMS tissue samples obtained from patients with LMA and/or LMS. Data were quantified using WinROOF Ver.6.3.0 software (Mitani Co., Ltd, Fukui, Japan). P vales were generated using a *t*-test. Data are representative of three experiments. +++: diffuse-positive (homogeneous distribution with more than 90% of cells stained), -: negative (no stained cells).

The non-standard subtypes of uterine mesenchymal tumors such as the epithelioid and myxoid types have been classified in a different manner using these features; therefore, a diagnostic method needs to be established that can identify non-standard smooth muscle differentiation [5, 6]. Pathological studies have been performed to demonstrate the validity and reliability of LMP2/β1i as a diagnostic biomarker when combined with other candidate molecules, such as cyclin E and calponin h1, which reportedly function as anti-oncogenic factors in human Ut-LMS. Pathological examinations revealed that the ability to induce the expression of LMP2/β1i and calponin h1 was markedly lower in human Ut-LMS tissues than in uterine LMA or a normal myometrium located in the same section, and the expression of cyclin E was markedly high in human Ut-LMS tissues only [13-16]. The histological findings of skeletal muscle and rectum lesions were consistent with metastatic Ut-LMS [13, 14]. Western blotting and RT-PCR experiments revealed that LMP2/β1i was expressed in a normal myometrium, but not in human Ut-LMS, and both findings strongly supported the pathological results [13, 14, 16, 17]. Although we previously demonstrated that the abnormal expression of the ovarian steroid receptors, Tp53, ki-67 and mutations in Tp53 were frequently associated with Ut-LMS, the defective expression of LMP2/β1i and calponin h1 appeared to be more characteristic of human Ut-LMS than these factors [14, 15, 18-20] (Fig. 1).

We are currently collaborating with several clinical research facilities to investigate the reliability and characteristics of LMP2/β1i as a diagnostic indicator. The histopathological characteristics of human uterine mesenchymal tumors including mitotically active leiomyoma, bizarre leiomyoma, lipoleiomyoma, undifferentiated endometrial sarcoma, epithelioid variant leiomyosarcoma, myxoid variant leiomyosarcoma, smooth muscle tumors of uncertain malignant potential (STUMP) and leiomyomatoid angiomatous neuroendocrine tumor (LANT) have already been summarized [21-24]. Therefore, clarifying the relationships between these factors and the development of human Ut-LMS, and identifying specific risk factors may lead to the development of new clinical treatments for this disease.
